# Short- and Long-Term Storage of Non-Domesticated European Mouflon (*Ovis aries musimon*) Cumulus–Oocyte Complexes Recovered in Field Conditions

**DOI:** 10.3390/ani14050807

**Published:** 2024-03-05

**Authors:** Letizia Temerario, Vincenzo Cicirelli, Nicola Antonio Martino, Alice Carbonari, Matteo Burgio, Lorenza Frattina, Giovanni Michele Lacalandra, Annalisa Rizzo, Maria Elena Dell’Aquila

**Affiliations:** 1 Department of Biosciences, Biotechnologies & Environment, University of Bari Aldo Moro, Strada per Casamassima km 3, 70010 Valenzano, Italy; nicola.martino@uniba.it (N.A.M.); mariaelena.dellaquila@uniba.it (M.E.D.); 2 Department of Veterinary Medicine, University of Bari Aldo Moro, km 3 Strada per Casamassima, 70010 Valenzano, Italy; vincenzo.cicirelli@uniba.it (V.C.); alice.carbonari@uniba.it (A.C.); matteo.burgio@uniba.it (M.B.); l.frattina@gmail.com (L.F.); giovannimichele.lacalandra@uniba.it (G.M.L.); annalisa.rizzo@uniba.it (A.R.)

**Keywords:** female mouflon, oocyte, field oocyte collection, EH holding, vitrification, in vitro maturation (IVM), bioenergetic–oxidative status

## Abstract

**Simple Summary:**

The European mouflon (*Ovis aries musimon*) is a non-domesticated and highly adaptable species. It is a descendant of the primitive domestic sheep (*Ovis aries*), with native populations inhabiting Sardinia and Corsica since the Neolithic age. In the 1950s, as a consequence of intense hunting and poaching, some animals were relocated to nearby islands (Giglio Island and others) as a conservation strategy to avoid extinction risks. Since then, this isolated way of life has served as a “time capsule” in which the Ovis aries musimon has maintained ancestral genetic traits no longer detectable in the current native Sardinian population. Our study findings report the application, in field conditions, of short- and long-term storage techniques of immature cumulus–oocyte complexes (COCs) which were then transported to the laboratory for in vitro maturation (IVM) and assessment of nuclear and cytoplasmic maturation. Both techniques mentioned above can be used for oocyte storage and in vitro culture. However, it is still necessary to optimize them, particularly when used in taxonomically related and endangered wild species for the preservation of ancestral genetic traits and to address the risk of extinction of native populations.

**Abstract:**

Reproductive biotechnologies can be used as a supporting tool, through gamete conservation and in vitro embryo production, in the preservation of invaluable and irreplaceable animal genetic resources. In the present study, immature mouflon cumulus–oocyte complexes (COCs) collected from ovariectomized female ovaries underwent short- or long-term conservation (24 h maintained in Earle’s/Hank’s (EH) medium or vitrification) under field conditions and afterwards transported to the laboratory where they were cultured for in vitro maturation (IVM) and assessed for oocyte meiotic competence and bioenergetic–oxidative status. Utilization of both storage techniques led to COC morphology preservation, as well as cumulus expansion and oocyte meiotic resumption after the IVM procedure. Quantitative bioenergetic–oxidative parameters were reduced in vitrified oocytes compared with EH ones. Immature COC storage needs to be optimized in both domesticated and non-domesticated sheep as a part of the strategy to avoid the loss of valuable genotypes of these animal species.

## 1. Introduction

Many non-domesticated animal species are at risk of extinction due to human activities, pollution, climate change, emerging diseases, and invasive species [1]. Reproductive biotechnologies, including artificial insemination (AI), embryo transfer (ET), in vitro fertilization (IVF), intracytoplasmic sperm injection (ICSI), somatic cell nuclear transfer (SCNT), generation of induced pluripotent stem cell (iPSCs), and cryopreservation of gametes and embryos with gene banking procedures, can be utilized in the conservation of these species and to counteract their numerical reduction as well as genetic disappearance. However, the development of suitable reproductive biotechnology protocols for use in non-domesticated animals is of greater importance because of the lower knowledge of reproductive anatomy, physiology, and endocrinology and limited number of available gametes to evaluate efficacy when using these technologies [1,2,3]. Therefore, the use of common and suitable taxonomically related animals can be useful in initial studies particularly to validate experimental protocols which will be subsequently applied to rare non-domesticated animals [1,3].

The European mouflon (*Ovis aries musimon*) is a non-domesticated and highly adaptable medium-sized ungulate [4]. Native populations have resided on the island of Sardinia (Italy) and Corsica (France) since the Neolithic Age and, in the last century, have been transferred across Europe [5]. However, during the 20th century, there was a marked reduction in native animal population quantity with only a few hundred animals remaining due to intense poaching and habitat loss as a consequence of the sheep farming expansion [6,7]. In the 1950s, to halt further reduction and possible extinction, some animals were relocated from Sardinia to Giglio and other islands, as a conservation strategy [7]. The island inhabitation of relocated animals served as a “time capsule” in which mouflons maintained ancestral genetic traits no longer detectable in the current native Sardinian population [7]. However, the relocation of mouflons to this island was challenging, due to the ecological/sociological competition with native species, mating with domestic sheep, damage to the present ecosystem and cultivated crops, and mouflons’ progressive numerical increase [4]. Procedures such as ovariectomy and orchiectomy surgery, performed under field conditions, can be used to control the animals’ overpopulation and avoid selective culling of mouflons [4]. 

The utilization of reproductive biotechnologies through gamete conservation including these procedures is a potential approach to preserve the invaluable and irreplaceable genetic resource of the mouflon, reducing the future impact of unfavorable factors and the risk of numerical and genetic mouflon extinction. In particular, the preservation of wild female germplasm is a promising biotechnological approach to conserve animal species [2]. However, the collection of in vivo matured oocytes is difficult in non-domesticated animals, because adequate and standardized ovarian stimulation protocols still do not exist to provide optimization of oocyte collections from preovulatory follicles in such animals [1,3]. Therefore, a more feasible approach is the collection under field conditions of reproductive material (ovaries, fragments of ovarian tissue or immature cumulus–oocyte complexes, COCs) performed after ovariectomy, animal culling, or unexpected mouflon death [3]. To achieve this, it is necessary to develop suitable short- and long-term storage strategies for the preservation of collected reproductive tissue during transfer from the collection sites to the specialized laboratories, which will ensure oocyte viability and functionality such as maintenance of meiotic arrest without loss of developmental competence, and will allow further successful laboratory procedures [8,9,10].

Short-term storage addresses the successful transport of oocytes from the collection sites to the specialized laboratories within 4 to 72 h. The success of the transport depends on several factors, such as medium composition, transport temperature, duration of storage, and animal species evaluated [9]. Most of the studies have been conducted with horse oocytes because the limited number of slaughterhouses for horses, and the need to transfer collected oocytes to ICSI laboratories for further successful in vitro manipulations required the development of protocols for short-term storage of immature horse oocytes compared to other species [8,10]. Efficacious storage techniques for immature equine oocytes, without loss of capacity to subsequently mature or develop into blastocysts, was accomplished by placing oocytes in a modified M199 medium with deprived meiosis inhibitors, named EH medium (Earle’s/Hank’s), for 16 to 18 h at room temperature [8,11] and for 2 days at 15 °C [12]. Other studies are reported in cattle, where storage at room temperature in EH medium for 10 h or in commercial media for 16 to 18 h resulted in preservation of immature COCs without negative effects on subsequent meiotic and developmental competence [13,14]. A study with pigs indicated that the inhibition of meiosis resumption was achieved for up to 3 days at room temperature in porcine follicular fluid or fetal calf serum, with subsequent development to the metaphase II stage after IVM followed by development to the blastocyst stage [15]. According to similar procedures in wild animals, we found only one study in Iberian red deer (*Cervus elaphus hispanicus*), where mature oocytes were developed after storage for 16 h in EH medium at room temperature [16].

The cryopreservation of oocytes in liquid nitrogen at −196 °C by slow freezing or vitrification techniques, coupled with the gene banking strategy, provides unlimited preservation of oocytes and their utilization in the near or distant future when there is the need to do so [17]. In particular, vitrification of immature COCs allows the avoidance of animal hormonal stimulation as an advantage for the field conditions and particularly wild animals. Moreover, importantly, the minimizing of necessary equipment for the procedure (stereomicroscope, cryoprotectant-rich solutions, vitrification devices, and liquid nitrogen) allows flexible scheduling of the IVM day, independent of COC collection and selection time [18]. On the other side, oocytes are among the most difficult cells to cryopreserve because of their high sensitivity to the mechanical, chemical, osmotic, and thermal stresses imposed during freezing/warming procedures [19]. In particular, preservation of physical and functional integrity between the cumulus cells and the oocyte is a major challenge for successful IVM from vitrified immature COCs. Although the maturation and percentage of development to the blastocyst stage continue to be less, compared to freshly collected oocytes, live births after these procedures have been reported in cattle [20], swine [21], horses [22,23], and domestic cats [24]. In wild animals, a study of the four-horned antelope chousingha (*Tetracerus quadricomis*) reported a lower rate of mature oocyte development after immature COC vitrification in comparison with freshly collected oocytes [25]. However, another study with a different wild animal species (African lion, *Panthera leo*) indicated a comparable percentage of immature oocytes developing to the MII stage but no development beyond the 4-cell embryonic stage [26].

**Figure 1:**
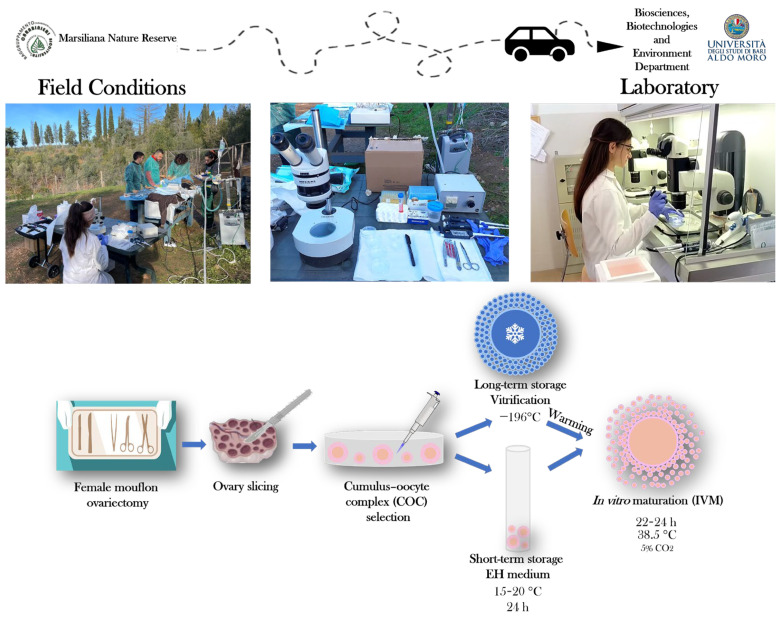
Schematic representation of the study’s experimental design. After ovariectomy under field conditions, female mouflon ovaries were processed by slicing to collect cumulus–oocyte complexes (COCs). Selected immature COCs were subjected to short-term (24 h storage in Earle’s/Hank’s medium, EH) or long-term (vitrification) storage for their preservation and transportation from the collection site to the IVM laboratory.

The aim of the present study was to compare and evaluate the oocyte developmental capacity of immature COCs collected from female mouflons after ovariectomy in field conditions, after their short-term storage in EH medium and long-term storage by vitrification. Following the preservation period, we evaluated and compared oocyte meiotic competence and bioenergetic–oxidative status after IVM. The summary of our experimental design is presented at Figure 1.

## 2. Materials and Methods

### 2.1. Ethics

This study was performed in agreement with the ethical guidelines of the Animal Welfare Committee. Institutional Review Board approval of the study was obtained from University of Bari “Aldo Moro” (n. 20/2022).

### 2.2. Chemicals

Unless otherwise indicated, chemicals were purchased from Sigma-Aldrich (Milan, Italy).

### 2.3. Ovary Processing and COC Retrieval

**Figure 2:**
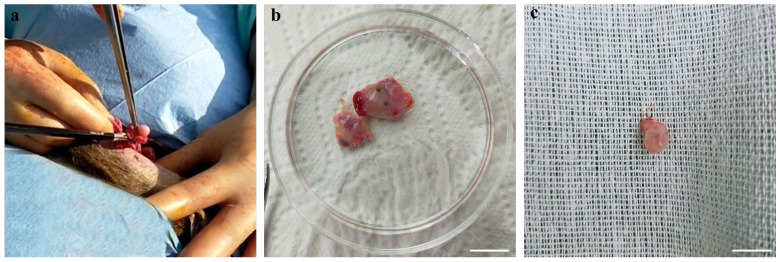
Female mouflon ovariectomy: (**a**) ovarian excising; (**b**) ovaries separated from an adult animal and (**c**) ovary from the prepubertal ewe. Scale bar represents 1 cm.

**Figure 3:**
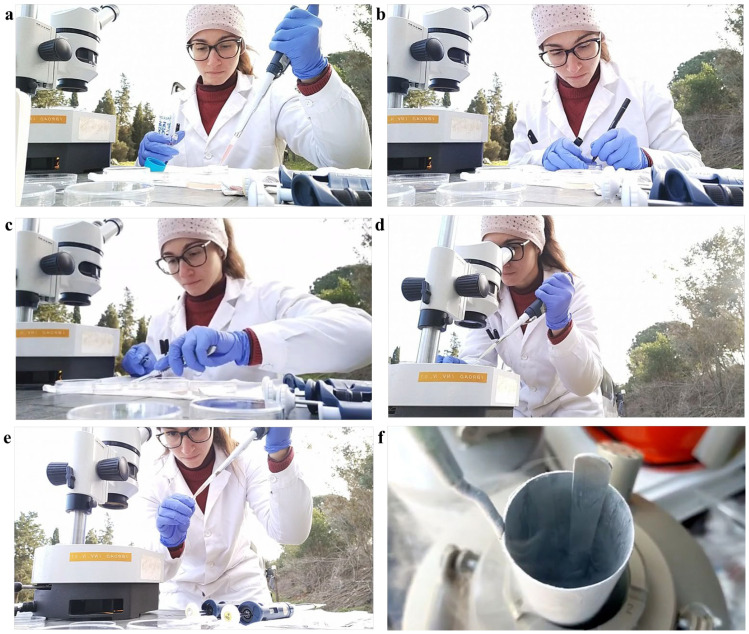
Short- and long-term storage of female mouflon cumulus–oocyte complexes (COCs): (**a**) preparation of the plate for COC selection; (**b**) labeling of the culture plate with sample (animal) number; (**c**) slicing procedure; (**d**) COC selection; (**e**) filling a glass vial with EH medium and selecting COCs for short-term storage; (**f**) long-term storage of vitrified COCs in one dewar filled with liquid nitrogen.

Ovaries were collected after the ovariectomy of female mouflons, from 10 animals between 18 and 28 months and one prepubertal animal of 5 months, all originating from Giglio Island and relocated to the Marsiliana Nature Reserve, Grosseto, Italy [4]. The ovaries were processed immediately after the ovariectomy in field conditions. For COC retrieval, ovaries were sliced [18] and follicular contents collected in sterile Petri dishes containing phosphate-buffered saline (PBS) and observed using a WILD Heerbrugg stereomicroscope. Only COCs displaying at least three intact cumulus cell layers and a homogenous cytoplasm were selected for short- or long-term storage and subsequent in vitro culture. The number of animals from which COCs were assigned to short- or long-term storage was determined based on the surgery timing: COCs recovered on the first day were vitrified while those collected on the second day of the surgery were assigned to EH processing since our team returned to the laboratory on the second day. The process of ovary retrieval is illustrated in Figure 2 where (a) shows ovarian excision, (b) shows the ovaries separated from the adult animal, and (c) shows the ovary from the prepubertal ewe. Collection of the pictures presented as Figure 3 were taken during the procedures used for COC collection and oocyte storage in field conditions.

### 2.4. Short-Term Storage of COCs in EH Medium 

Ovaries collected from three of the adult females were assigned to the EH medium treatment and placed in 1 mL of medium consisting of a 40% (*v*/*v*) Earle’s and 40% (*v*/*v*) Hank’s salts-buffered M199 mixture with 20% fetal calf serum (FCS) and 25 μg/mL gentamicin in 8 mm × 40 mm glass vials (Thermo Scientific, Waltham, MA, USA), under field conditions [8,11,12]. The vials were cap sealed and protected from daylight exposure by storage in aluminum foil. The COCs were transported to the laboratory at a temperature of 15- to 20 °C and further processed within 24 h after collection. In the IVM laboratory, EH-treated COCs were first morphologically evaluated and then transferred into an IVM culture medium. 

### 2.5. Long-Term Storage of COCs by Vitrification

COCs from other adult animals as well as from the prepubertal female were cryopreserved using the previously established vitrification technique for ovine COCs [18]. Briefly, selected immature COCs were incubated for 10 min in a 300 μL drop of equilibration solution (ES) consisting of HEPES-buffered TCM 199 medium supplemented with 20% (*v*/*v*) FCS (base medium, BM) containing 7.5% (*v*/*v*) ethylene glycol (EG) and 7.5% (*v*/*v*) dimethyl sulfoxide (DMSO). After equilibration, the oocytes were transferred in less than 60 s into a 300 μL drop of vitrification solution (VS) consisting of BM containing 15% (*v*/*v*) EG, 15% (*v*/*v*) DMSO, and 0.5 mol/L sucrose. Oocytes were subsequently transferred into an Open Pulled Straw (OPS) (Minitube, Tiefenbach, Germany) with a minimum volume (e.g., <0.1 μL). The described procedure was performed at external ambient temperature under field conditions (5- to 10 °C). At the end, samples were quickly plunged into liquid nitrogen at −196 °C. 

### 2.6. Warming of Vitrified COCs

Vitrified COCs were transported in liquid nitrogen at −196 °C to the IVM laboratory where warming and IVM were performed [18]. The vitrified devices were directly submerged into one 100 µL drop of warming solution (WS) consisting of BM containing 1 mol/L sucrose at 38.5 °C for 1 min. Warmed oocytes were transferred to a 300 μL drop of dilution solution (DS) consisting of BM with 0.5 mol/L sucrose for 3 min and then washed twice in 300 μL drops of BM for 5 min. After warming, vitrified COCs were morphologically evaluated and placed in IVM culture. 

### 2.7. Assessment of COC Morphology after Short- or Long-Term Storage

**Figure 4:**
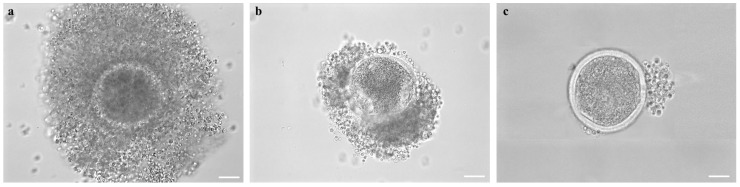
Female mouflon COC morphologies as observed after short- or long-term storage: (**a**) example of COC with “preserved morphology”, displaying complete and compact cumulus with cells in close contact with each other and with the oocyte; (**b**) COC with a cumulus partially detached from the zona pellucida; (**c**) Denuded COC with all the cumulus cells completely detached. Scale bar represents 40 µm.

COC morphology, after short- or long-term storage, was evaluated. The COCs were classified as (a) “preserved morphology” if displaying complete and compact cumulus with cells in close contact with each other and with the oocyte; (b) “partially removed cumulus”, if cumulus cells were partially detached from the oocyte, and finally (c) “completely removed cumulus”, if cumulus cells were completely detached and, in some cases, when the oocyte structure was completely impaired due to discontinued integrity of the zona pellucida (Figure 4).

### 2.8. In Vitro Maturation (IVM)

IVM methods were adapted from those previously described for domestic sheep (*Ovis aries*) [18]. The IVM medium was prepared based on TCM-199 medium with Earle’s salts, buffered with 5.87 mmol/L HEPES and 33.09 mmol/L sodium bicarbonate and supplemented with 0.1 g/L L-glutamine, 2.27 mmol/L sodium pyruvate, calcium lactate pentahydrate (1.62 mmol/L Ca^2+^, 3.9 mmol/L Lactate), 50 μg/mL gentamicin, 20% (*v*/*v*) fetal calf serum (FCS), 10 μg/mL of porcine follicle stimulating hormone and luteinizing hormone (FSH/LH; Pluset^®^, Calier, Barcellona, Spain) [27], and 1 μg/mL 17β estradiol [18]. The prepared medium was pre-equilibrated for 1 h under 5% CO_2_ in air at 38.5 °C, then transferred (400 μL/well) into a four-well dish (Nunc Intermed, Roskilde, Denmark) and covered with pre-equilibrated lightweight paraffin oil. The COCs from each animal were cultured individually in one well of a four-well dish for 22- to 24 h at 38.5 °C in an atmosphere containing 5% CO_2_ in air.

### 2.9. Assessment of Cumulus Expansion and Oocyte Denuding

After IVM, COCs were collected and cumulus expansion evaluated. The COCs with continuous edges of cumulus cells in close contact with each other and with the oocyte were classified as compact, whereas cumuli with discontinuous edges of cells detached from each other and immersed in a viscous extracellular matrix were classified as expanded. Oocyte denuding was performed by incubation in TCM-199 with 20% FCS containing 80 IU hyaluronidase/mL. Denuded oocytes were then evaluated for meiotic stage development and bioenergetic/oxidative status.

### 2.10. Oocyte Mitochondria and ROS Staining

To detect and localize ooplasmic mitochondria and reactive oxygen species (ROS), oocytes underwent staining with MitoTracker Orange CMTM Ros (Thermo Fisher Scientific, Waltham, MA, USA) and 2,7-dichlorodihydrofluorescein diacetate (H_2_DCF-DA), and fixation in 4% paraformaldehyde (PFA) solution in PBS [18,28]. 

### 2.11. Oocyte Nuclear Chromatin Evaluation

Oocyte nuclear chromatin was evaluated after fixation in 4% PFA in PBS, staining with 2.5 µg/mL Hoechst 33258 in 3:1 (*v*/*v*) glycerol/PBS and mounting on microscope slides. Slides were examined by using an epifluorescence microscope (Nikon Eclipse TE300; Nikon Instruments, Firenze, Italy) equipped with the objective Nikon Plan Fluor 40×/NA 0.75 and a B-2A (346 nm excitation/460 nm emission) filter. Oocytes were classified as immature/abnormal (including germinal vesicle, multipolar meiotic spindle, irregular chromatin clumps, or the absence of chromatin), metaphase I to telophase I (MI to TI) and metaphase II (MII) with the first polar body (PB) extruded [18]. 

### 2.12. Assessment of Mitochondrial Distribution Pattern and Intracellular ROS Localization

Oocytes were observed using a Nikon C1/TE2000-U laser scanning confocal microscope (Nikon Instruments, Firenze, Italy) equipped with the objective Nikon Plan Apo 60×/NA 1.40 in oil immersion. A 543 nm helium/neon laser and a G-2A filter were used to detect the MitoTracker Orange CMTM Ros (551 nm excitation and 576 nm emission). A 488 nm argon ion laser and a B-2A filter were used to detect dichlofluorescein (DCF) (495 nm excitation and 519 nm emission). To allow 3D distribution analysis, oocytes were observed in 25 optical sections with a step size of 0.45 µm. The mitochondrial distribution pattern was evaluated using previously reported criteria: (1) finely granular, typical of immature oocytes; (2) perinuclear and subcortical (P/S), indicating cytoplasmic maturity; and (3) abnormal, displaying irregular distribution of mitochondria [18]. Oocytes with intracellular ROS diffused throughout the cytoplasm, together with areas/sites of mitochondria/ROS overlapping, were considered viable.

### 2.13. Quantification of Bioenergetic/Oxidative Variables

In each individual oocyte, MitoTracker and DCF fluorescence intensities and the Manders’ overlap coefficient [29], indicating the extent of mitochondria/ROS colocalization, were measured at the equatorial plane using the EZ-C1 Gold Version 3.70 image analysis software platform for a Nikon C1 confocal microscope. A circular area was drawn to measure only the region including cell cytoplasm. The fluorescence intensity within the scanned area (512 × 512 pixels) was recorded and 16-bit images were obtained. Mitochondrial membrane potential (ΔΨ) and intracellular ROS concentrations were recorded as the fluorescence intensity emitted by each probe and expressed as arbitrary densitometric units (ADUs). Sample signals were expressed as percentages of the signal of the sample used as a control (EH treatment). Variables related to fluorescence intensity, such as laser energy, signal detection (gain), and pinhole size, were maintained at constant values for all measurements. In mitochondria/ROS colocalization analysis, threshold levels were kept constant, for all measurements, at 10% of the maximum pixel intensity.

### 2.14. Statistical Analysis

The proportions of COCs with different morphologies after short- or long-term storage and IVM, and the values for oocytes having different chromatin characteristics (immature/abnormal, MI to TI and MII + PB) and mitochondria distribution patterns were compared between groups using the Χ^2^ test. Quantification data, i.e., ΔΨ, intracellular ROS concentrations and mitochondria/ROS colocalization, were expressed as mean ± standard deviation (s.d.) and were compared using the unpaired Student’s *t*-test. There were considered to be mean differences when there was a *p* < 0.05 value.

## 3. Results

### 3.1. Short- and Long-Term Storage Preserved Oocyte Meiotic Competence in Mouflons

**Figure 5:**
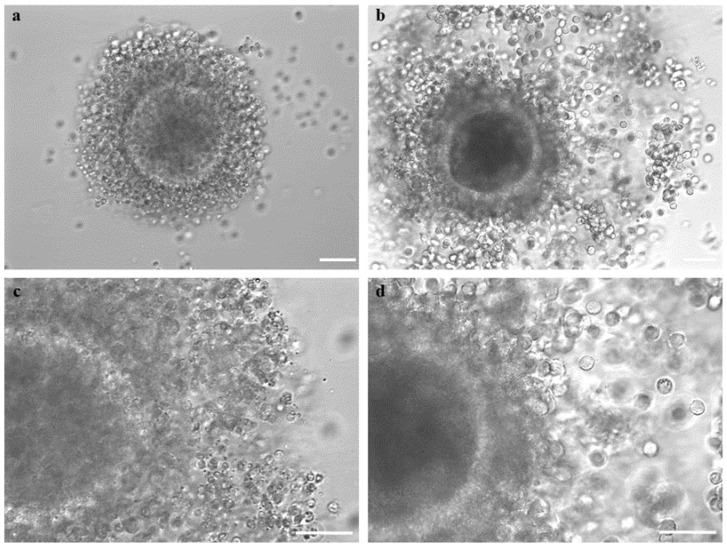
Female mouflon COC after vitrification/warming as observed (**a**,**c**) before and (**b**,**d**) after IVM. Before IVM, COC had a complete, compact, and multilayered cumulus. After IVM, there was regular cumulus expansion, with individually visible cumulus cells and cytoplasmic protrusions. Scale bars represent 40 μm.

In adult female mouflons, ovary size ranged between 1.3 ± 0.4 cm and 1.1 ± 0.2 cm for the longitudinal and transverse diameter, respectively. The ovaries contained follicles in different developmental stages, from 1 to 5 mm. Some ovaries contained corpora lutea, indicating the diestrus phase of the reproductive cycle. Contrary, in other ovaries, corpora lutea were absent indicating that these females were in the proestrus phase of the estrous cycle (Figure 2). The values for ovary sizes of the only prepubertal ewe lamb were 0.8 ± 0.1 and 0.6 ± 0.1 cm for the longitudinal and transverse diameter, respectively, and presented follicles in different developmental stages, from 1 to 3 mm. Oocyte recovery rates ranged from 1 to 15 good-quality COCs per donor, both in adult and prepubertal female mouflons. In total, we recovered 23 COCs from three female adult donors (subjected to EH treatment), and 26 COCs from seven other adults (*n* = 21) and the only prepubertal female (*n* = 5) (subjected to vitrification) (Figure 3). The COC morphology was preserved after storage (complete compact and multilayered cumuli as observed in freshly recovered COCs), regardless of the method used (EH or vitrification) (Table 1; Figure 4). After the IVM procedure, the initial impression was that cumulus expansion of immature vitrified COCs was in a greater percentage compared with EH COCs, but these values were not different when evaluated by statistical tools (Table 2; Figure 5). After IVM culture, COCs resumed meiosis and developed to the metaphase II stage without differences between short- and long-term storage (Table 3). 

### 3.2. Short- and Long-Term Storage Did Not Affect Mitochondria Distribution Pattern in Mouflon Oocytes

**Figure 6:**
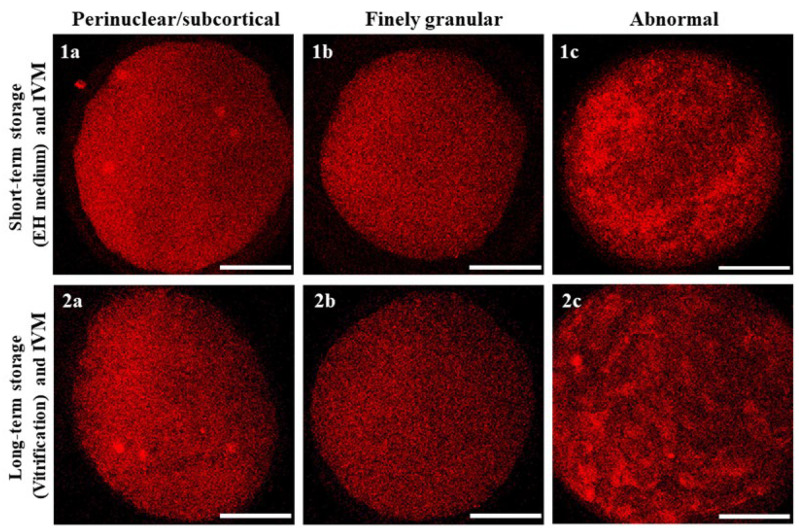
Oocyte mitochondrial distribution patterns classified as (**1a**,**2a**) perinuclear/subcortical, (**1b**,**2b**) finely granular and (**1c**,**2c**) abnormal, as observed after short- or long-term storage, IVM and staining with MitoTracker Orange. Scale bars represent 40 µm.

Regardless of the oocyte storage technique utilized, the majority of matured (MII + PB) oocytes had finely granular mitochondrial distribution patterns, with small mitochondria aggregates spread throughout the cytoplasm and no detectable differences between the two storage methods. This was also the case for oocytes which developed to the MI to TI stages. Instead, oocytes that did not resume maturation or did so abnormally (immature/abnormal) had finely granular but also abnormal mitochondrial distribution patterns consisting of large clumps of mitochondria irregularly distributed throughout the cytoplasm (Table 4; Figure 6).

### 3.3. Vitrification Affects Quantitative Bioenergetic Parameters in Mouflon Oocytes 

**Figure 7:**
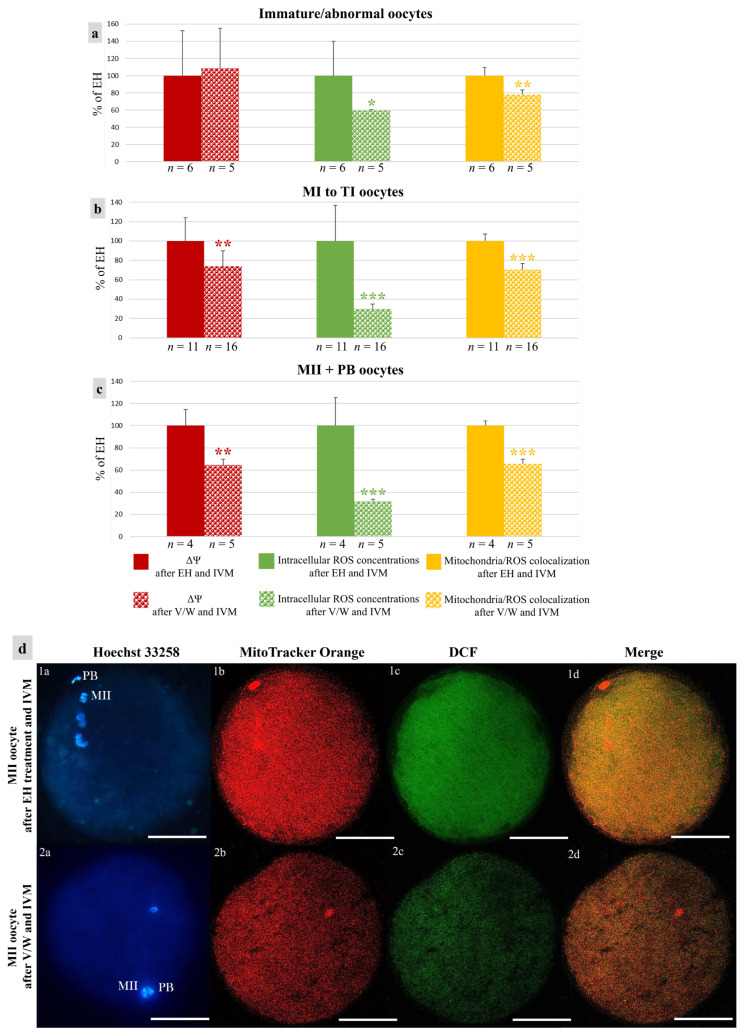
Effects of short- or long-term storage of mouflon COCs on oocyte bioenergetic/oxidative variables after IVM: (**a**–**c**) Means ± standard deviations of ΔΨ, intracellular ROS concentrations and mitochondria/ROS colocalization in mouflon oocytes. Values of vitrified/warmed (*v*/*w*) oocytes are presented as a percentage of the signal of oocytes after EH treatment and IVM. Number of oocytes per chromatin configuration is indicated at the bottom of each bar. Unpaired Student’s *t*-test: * *p* < 0.05; ** *p* < 0.01; *** *p* < 0.001. (**d**) Photomicrographs of mature oocytes after short- or long-term storage and IVM. Corresponding epifluorescence images of (**1a**,**2a**) nuclear chromatin characteristics and confocal images of (**1b**,**2b**) mitochondrial distribution pattern and activity, (**1c**,**2c**) intracellular ROS localization and concentrations and (**1d**,**2d**) mitochondria/ROS colocalization. Images taken at oocyte equatorial plane. Scale bars represent 40 µm.

In vitrified–warmed immature/abnormal oocytes, ROS levels and mitochondria/ROS colocalization were reduced in comparison with those of EH oocytes (*p* < 0.05 for intracellular ROS concentrations and *p* < 0.01 for the Manders’ overlap coefficient, Figure 7a). As well, in vitrified–warmed oocytes at MI-TI and MII + PB stages, all three quantitative bioenergetic parameters were reduced (*p* < 0.01 for ΔΨ, *p* < 0.001 for intracellular ROS concentrations, and *p* < 0.001 for the Manders’ overlap coefficient, Figure 7b,c). 

## 4. Discussion

The utilization of reproductive biotechnologies is an efficient approach for in situ conservation of endangered animal species and breeds against genetic concentration and extinction risk, even in extreme conditions [18]. The case of the Giglio Island mouflons is particularly emblematic, because results from recent studies highlighted the need to safeguard the genetic uniqueness of this nucleus of animals for which isolation on the island led to a “time capsule” effect that preserved ancestral genetic traits no longer detectable in the current native Sardinian population [7]. For this reason, among other measures, it is important to develop gamete conservation protocols to safeguard the genetic diversity of this mouflon population, as a sort of “insurance” against current and future events that might compromise the sustenance of mouflon as a species. To the best of our knowledge, only results from one study have been published for this species, designed for the development of a mouflon gene bank [6]. This study reported data about semen cryopreservation, in vitro produced embryos, and granulosa cells but lacked data for any procedure with oocyte involvement. 

In our study, short- and long-term preservation strategies for immature COCs in mouflons were conducted for the first time. Vitrification is the optimal method for preserving COCs for long-term purposes. However, the results in domestic sheep are still limited, requiring optimization of these technique for wider use in various sheep species and breeds [18,30,31,32,33]. The evaluation of the short-term storage in EH medium procedure was considered interesting for our research group because this is the only approach that allows the use of fresh and non-cryopreserved samples. The short-term storage procedure of immature COCs in EH medium has previously been reported for oocytes of other animal species [8,10,11,12,13,14,15,16] and in the present study was evaluated for the first time in the genus Ovis.

Although the EH technique gave positive results in the preservation of the physical integrity and intercellular contacts of oocytes and cumulus cells, it did not result in cumulus expansion. Indeed, even though cumulus expansion does not fully ensure the achievement of oocyte maturation, it represents an important aspect of COCs’ response to the gonadotropins added to the culture medium. We hypothesized that the metabolic requirements for these biological processes differ among species, and further studies could be directed toward modifications of EH and IVM medium composition for successful use in the preservation of mouflon COCs. On the other hand, a COC vitrification technique led to more encouraging results, represented by an increased percentage of COCs with a preserved morphology after *v*/*w* and an expanded cumulus. 

Oocyte nuclear maturation evaluation showed no differences in the percentage of oocytes that resumed meiosis and developed to the metaphase II stage when EH and vitrification technologies were used, with similar values around 20%. These results are novel since in the previous study conducted on mouflons, only IVC evaluations were performed, followed by embryo transfer, without including oocyte IVM data [6]. Furthermore, these data cannot be compared with those for immature COCs of domestic sheep subjected to IVM immediately after retrieval. In the present study, there was evaluation of EH-COCs and not of freshly collected COCs because the study was conducted in field conditions where it was necessary to, as a minimum, utilize a short-term storage strategy. The results from the present study are encouraging for the development of animal germplasm conservation strategies, because 20% of mature oocytes may be important for the implementation of these strategies.

Furthermore, when bioenergetic–oxidative status was evaluated in our study, the mitochondrial distribution pattern was finely granular, displaying small mitochondria aggregates throughout the cytoplasm, regardless of the oocyte meiotic stage and the technique utilized. In domestic ovine species this pattern is typically associated with cytoplasmic immature oocytes [18]. Still, an evaluation of the mitochondrial distribution pattern of in vivo matured oocytes in this species is still lacking. Regardless of meiotic stage, intracellular ROS concentrations and mitochondria/ROS colocalization of vitrified oocytes were less compared to those of EH oocytes. ΔΨ was also reduced in oocytes at the MI-TI and MII + PB meiotic stages. These findings could be explained by considering that the control values in this study belong to EH oocytes which may have displayed higher oxidative stress compared with fresh ones.

## 5. Conclusions

In conclusion, we report for the first time the application of ex situ conservation strategies for the female germplasm of the non-domesticated European mouflon, through short- and long-term storage of immature COCs obtained after ovariectomy in field conditions. Immature COC preservation in EH medium and cryopreservation by vitrification procedure are strategies that have the potential to be utilized to effectively preserve the viability, integrity, and functionality of the COC. This was indicated by relatively high/satisfactory values of COCs with preserved morphology after storage, expansion of the cumulus oophorus, meiosis resumption and progressive development to the metaphase II stage. However, there is a need to optimize short- and long-term storage, first in taxonomically related domestic species with subsequent application and adaption of the established procedure on non-domesticated animals. In this way, we can achieve increasing results in the meiotic and developmental competence of these oocytes, as an “insurance” to avoid the extinction of endangered species in the case of unpredictable detrimental occurrences that affect species sustainability.

## Figures and Tables

**Table 1:** Effects of short- (EH) or long-term (vitrification) storage on cumulus–oocyte complex (COC) morphology in mouflons.

COC Treatment	N. of Healthy Selected COCs (N. of Donors)	COC Morphology after Short- or Long-Term Storage N. (%)		
		Preserved	Partially Removed Cumulus	Completely Removed Cumulus
EH	23 (3)	15 (65.2)	4 (17.4)	4 (17.4)
Vitrification	26 (8)	21 (80.8)	4 (15.4)	1 (3.8)

Table legend: COC = Cumulus–oocyte complex; Χ^2^ test: within each column, comparisons between EH and vitrified COCs: not significant.

**Table 2:** Effects of short- (EH) or long-term (Vitrification) storage on COC expansion after IVM in mouflons.

COC Treatment	N. of Cultured COCs (N. of Donors)	Cumulus Expansion after Short- or Long-Term Storage and IVM N. (%)		
		Yes	No	Removed
EH	23 (3)	2 (8.7)	12 (52.2)	9 (39.1)
Vitrification	26 (8)	9 (34.6)	14 (53.8)	3 (11.5)

Table legend: COC = Cumulus–oocyte complex; Χ^2^ test: within each column, comparisons between EH and vitrified COCs: not significant.

**Table 3:** Effects of short- (EH) and long-term (Vitrification) storage on oocyte maturation rate after IVM in mouflons.

COC Treatment	N. of Cultured COCs (N. of Donors)	N. of Evaluated Oocytes	Nuclear Chromatin Characteristics N. (%)		
			Immature/ Abnormal	MI to TI	MII + PB
EH	23 (3)	21	6 (28.6)	11 (52.4)	4 (19.0)
Vitrification	26 (8)	26	5 (19.2)	16 (61.5)	5 (19.2)

Table legend: COC = Cumulus–oocyte complex; M = Metaphase; PB = Polar body. Χ^2^ test: within each column, comparisons between EH and vitrified COCs: not significant.

**Table 4:** Effects of short- (EH) or long-term (Vitrification) storage and IVM on oocyte mitochondria distribution pattern in mouflons.

COC Treatment	Meiotic Stage	N. of Evaluated Oocytes	Mitochondrial Distribution Pattern N. (%)		
			P/S	Finely Granular	Abnormal
EH	Immature/ Abnormal	6	0 (0)	5 (83.3)	1 (16.7)
Vitrification	Immature/ Abnormal	5	0 (0)	2 (40.0)	3 (60.0)
EH	MI-TI	11	1 (10.0)	10 (90.0)	0 (0)
Vitrification	MI-TI	16	2 (12.5)	13 (81.3)	1 (6.3)
EH	MII + PB	4	1 (25.0)	3 (75.0)	0 (0)
Vitrification	MII + PB	5	1 (20.0)	3 (60.0)	1 (20.0)

Table legend: M = Metaphase; PB = Polar body; P/S = Perinuclear/subcortical. Χ^2^ test: within each column, comparisons between oocytes after EH treatment or vitrification and IVM, for each meiotic stage: not significant.

## Data Availability

The data presented in this study are available on request from the corresponding author.
